# Impact of Social Distancing on Individuals Who Use Drugs: Considerations for Emergency Department Providers

**DOI:** 10.5811/westjem.2020.7.47896

**Published:** 2020-08-18

**Authors:** Kathy T. LeSaint, Hannah R. Snyder

**Affiliations:** * University of California San Francisco, Department of Emergency Medicine, San Francisco, California; † California Poison Control System, San Francisco, California; ‡ University of California San Francisco, Department of Family and Community Medicine, San Francisco, California

## Abstract

The isolation that comes from social distancing during the COVID-19 pandemic can be particularly detrimental to the United States’ population of people who use drugs. People with substance use disorders may be at risk for return to use, exacerbation of existing mental health disorders, and risky drug practices. In this commentary, we review the risk to people who use drugs and how emergency department providers can best support these individuals during the unprecedented time of social distancing.

On January 20, 2020, the first confirmed case of coronavirus disease 2019 (COVID-19), caused by the SARS-CoV-2 virus, was reported in the United States (US). As of April 16, 2020, the US death toll was at 29,998, exceeding that of any other country in the world.^1^ While the research community is working tirelessly to determine the most efficacious treatments for COVID-19, public health officials announced aggressive recommendations in hopes of slowing human-to-human spread. On March 19, 2020, California became the first state to mandate shelter in place, and New York was quick to follow. By April 7, 2020, 42 states, along with a number of cities and counties, had urged residents to stay in their homes except for essential trips and services. Whether under a shelter in place, safer at home, or stay home order, the concept of social distancing is strongly encouraged, defined by the US Centers for Disease Control and Prevention (CDC) as “keeping space between yourself and other people.”^2^ Social distancing is essential for community health but may be uniquely challenging for people who use drugs (PWUD) to comply with, and may put them at risk for drug-related harms.

## Risks to People Who Use Drugs

Approximately one third of PWUD seeking treatment for substance use disorders are unhoused or live in congregate settings including residential treatment facilities, shelters, and single-room occupancy hotels.^3^ In these settings, following the CDC guidance on social distancing may be difficult or impossible. This issue has led to COVID-19 clusters in some homeless shelters and has led several communities to seek alternative housing in hotels for people experiencing homelessness.^4^,^5^

Those PWUD who have the ability to practice social distancing may face an increased risk of drug-related harms. Coping with isolation and health threats may lead to increased stress and anxiety in a vulnerable population already stricken with trauma and mental health issues. Social isolation can act as a trigger and is strongly correlative with mood and substance use disorders.^6^ These factors can exacerbate existing substance use as patients self-treat psychiatric symptoms or lead people with a history of substance use disorder to return to use.

For those who are actively using drugs, practicing harm reduction can be difficult in the setting of COVID-19. Traditional guidance is for PWUD to use with another person so that if they overdose, 911 can be called and naloxone can be used, but by following social distancing PWUD are unable to do this. As borders close and supply chains are disrupted, PWUD may seek out drugs from places other than their usual, trusted sources and thereby be at greater risk of exposure to an adulterated or contaminated supply. In addition, many needle and syringe exchange programs changed their models in response to the COVID-19 pandemic, either closing down completely or being unable to provide their typical services and programs (eg, referral to treatment programs, harm reduction education, naloxone distribution and education, etc). Such changes place PWUD at greater risk for unsafe practices and increased risk of communicable infectious diseases, skin and soft tissue infections, and drug overdose and death.

Furthermore, maintaining access to treatment and recovery services during a time of social distancing mandates is difficult. Throughout the country, support groups have been cancelled, treatment programs are limiting new patients, and inpatient treatment centers have limited visits.^6^–^8^ Opioid treatment programs, in which most patients rely on daily dispensing of medications to treat opioid use disorder, may have reduced access as well. Such changes make it harder for patients to newly access treatment and present challenges for those who are already in treatment. Without ease of access to places of recovery and medication-assisted treatment, patients are at risk of serious medical and psychological complications.

Fortunately, the federal government has recently made several changes to increase access to life-saving addiction treatment. The Centers of Medicare & Medicaid Services has loosened regulations and is compensating for telemedicine services, the Drug Enforcement Agency now supports telephone and audiovisual buprenorphine prescribing, and the Office of Civil Rights at the Department of Health and Human Services approved usage of popular apps to provide telehealth without risk of penalties for noncompliance with HIPAA.^9^–^11^ In addition, opioid treatment programs are providing longer durations of take-home doses of medications for treatment of opioid use disorder.^12^

## Emergency Department Support for People Who Use Drugs

It is likely that emergency departments (ED) across the US will see an increase in the number of PWUD experiencing withdrawal, experiencing overdose, or seeking treatment for their substance use disorder. Preliminary data from the ED at San Francisco’s only public hospital revealed a near twofold increase in the number of patients presenting with the chief complaint of “drug overdose” in March 2020 (67 patients/month; 1.2% of all ED encounters) when compared to averaged data from the prior six months (38 patients/month; 0.6% of all ED encounters). In addition, more than 35 states have reported increases in cases of opioid-related overdose and mortality.^13^ Therefore, in this unprecedented time of social distancing, emergency providers are placed in an additional frontline role of delivering patient-centered care for a highly at-risk population of PWUD.

Emergency clinicians should provide compassionate, evidence-based care to PWUD. Establishing rapport and motivational interviewing can be difficult in a time of enhanced precautions and extra personal protective equipment. However, continuing to take the time to speak in a non-stigmatizing way is vital in the therapeutic process and is the start to effective treatment for PWUD.^14^

In recent years, the practice of ED initiation of buprenorphine has rapidly become the standard of care.^15^ We encourage emergency clinicians to offer buprenorphine to any patients presenting with opioid use disorder.^16^ DATA 2000 waivers are not required to administer buprenorphine in the ED. First doses of buprenorphine can be rapidly administered in the ED, and patients should be linked to ongoing treatment. In addition, while it is not the usual practice of the emergency clinician to provide long-term medication prescriptions, in this unique time we encourage providers with DATA 2000 waivers to offer longer durations of buprenorphine prescriptions (up to 28 days) to appropriate patients. During the COVID-19 pandemic, it is more essential than ever that emergency clinicians provide this service and while doing so, receive institutional support that is much needed to overcome barriers to buprenorphine administration. Individual institutions and departmental leadership can best support their clinicians by providing adequate training and resources regarding buprenorphine use, as well as assisting providers in coordination of outpatient linkage to care.^17^

On March 19, 2020, the Substance Abuse and Mental Health Services Administration provided additional guidance for managing the treatment of alcohol or benzodiazepine withdrawal in acute settings.^18^ Providing buprenorphine to treat patients with opioid use disorder and medication treatment for alcohol withdrawal is particularly essential for those patients who are diagnosed with COVID and entering quarantine. Adequately treated withdrawal and compassionate care will support them in staying for the duration of their quarantine period.

As much as possible, emergency care providers must continue to offer harm reduction strategies to PWUD. Strategies of harm reduction include supporting drug use hygiene (eg, giving education on safe consumption, distributing pipes or syringes), providing overdose prevention supplies (eg, take-home or prescriptions of naloxone, fentanyl test strips), and encouraging patients to not use drugs in isolation (eg, video-chatting with a buddy, contacting support at www.neverusealone.com). Involving an ED social worker or substance use navigator who is familiar with local outpatient resources and/or changes to the outpatient landscape during this time can help facilitate linkage to care. Finally, continuing to address other social determinants of health (eg, housing insecurity, psychiatric illnesses) is paramount to providing safe discharge to the community.

## CONCLUSION

The isolation that comes from social distancing during the COVID-19 pandemic can be particularly detrimental to the population of people who use drugs. People with substance use disorders may be at risk for return to use, exacerbation of existing mental health disorders, and risky drug practices. In this time, emergency care providers have a vital role in supporting this vulnerable population of people who use drugs by establishing rapport, encouraging best practices in harm reduction, providing medication treatment, and connecting patients to outpatient resources.

## References

[1] Johns Hopkins University and Medicine (2020). Coronavirus Resource Center. https://coronavirus.jhu.edu/us-map.

[2] Centers for Disease Control and Prevention Social distancing, quarantine, and isolation.

[3] Eyrich-Gerg KM, Cacciola JS, Carise D (2008). Individual characteristics of the literally homeless, marginally housed, and impoverished in a US substance abuse treatment-seeking sample. Soc Psychiatry Psychiatr Epidemiol.

[4] Mosites E, Parker EM, Clarke KE (2020). Assessment of SARS-CoV-2 infection prevalence in homeless shelters – four US cities, March 27–April 15, 2020. MMWR Morb Moral Wkly Rep.

[5] Frandino N, Stapleton S, Paul K (2020). Coronavirus forces San Francisco to put homeless into hotels.

[6] Chou KL, Liang K, Sareen J (2011). The association between social isolation and DSM-IV mood, anxiety, and substance use disorders: Wave 2 of the National Epidemiologic Survey on Alcohol and Related Conditions. J Clin Psychiatry.

[7] Cenziper D, Brulliard K, Jacobs J People in addiction treatment are losing crucial support during coronavirus pandemic.

[8] Bebinger M (2020). Opioid addiction is “a disease of isolation,” so pandemic puts recovery at risk.

[9] Centers for Medicare & Medicaid Services Medicare Telemedicine Health Care Provider Fact Sheet.

[10] Prevoznik TW DEA-068 letter.

[11] US Department of Health & Human Services Notification of Enforcement Discretion for Telehealth Remote Communications During the COVID-19 Nationwide Public Health Emergency.

[12] Substance Abuse and Mental Health Services Administration Opioid Treatment Program (OTP) Guidance.

[13] American Medical Association Issue brief: reports of increases in opioid-related overdose and other concerns during COVID pandemic.

[14] Clement S, Schauman O, Graham T (2015). What is the impact of mental health-related stigma on help-seeking? A systematic review of quantitative and qualitative studies. Psychol Med.

[15] Strayer RJ, Hawk K, Hayes BD (2020). Management of opioid use disorder in the emergency department: a white paper prepared for the American Academy of Emergency Medicine. J Emerg Med.

[16] Initiating buprenorphine treatment in the emergency department.

[17] Im DD, Chary A, Condella AL (2020). Emergency department clinicians’ attitudes toward opioid use disorder and emergency department-initiated buprenorphine treatment: a mixed-methods study. West J Emerg Med.

[18] Substance Abuse and Mental Health Services Administration (SAMHSA) (2015). Considerations for crisis centers and clinicians in managing the treatment of alcohol or benzodiazepine withdrawal during the COVID-19 epidemic: March 19, 2020. CNS Drugs.

